# Pictorial essay: USG of lumps and bumps of the foot and ankle

**DOI:** 10.4103/0971-3026.63048

**Published:** 2010-05

**Authors:** Rajesh Botchu, Aman Khan, Raj Bhatt

**Affiliations:** Department of Musculoskeletal Radiology, University Hospital of Leicester, Leicester, UK

**Keywords:** Foot, lumps, sonography

## Abstract

USG is a cost-effective and dynamic way to interrogate superficial lumps and bumps. We present a pictorial review of the USG findings in various “lumps and bumps” of the foot and ankle.

## Introduction

The use of musculoskeletal USG has increased tremendously over the last decade. USG is easily accessible and cheap. It helps to evaluate a lump in real time. The subcutaneous tissue, tendons, plantar fascia, synovium, and ligaments can be assessed with dynamic USG.[1–5] Familiarity with normal ultrasound appearances and knowledge of anatomy and pathological conditions are the keys to accurate assessment of normal variants and pathological conditions. The use of color and power Doppler enables one to assess the vascularity of lesions. USG can be used in acute, subacute, and chronic conditions.[6] Comparison with the contralateral foot and ankle can help one to identify normal variants and evaluate a lump. Although ultrasound cannot be a replacement for MRI, we feel that it should be the first line of investigation for a lump or bump in the foot. We present the characteristic USG findings of lumps and bumps of the foot and ankle.

## Methodology

**Figure 1 (A,B):**
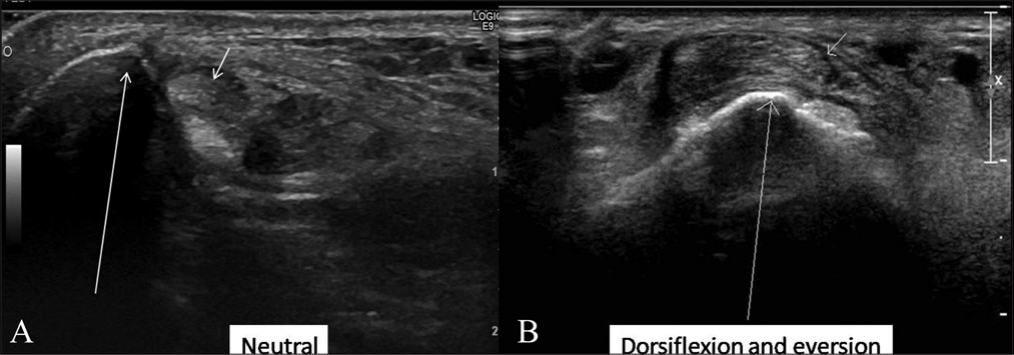
Subluxed peroneal tendons- Transverse USG in neutral position (A) and in dorsiflexion and eversion (B) shows normal position of the peroneal tendons (short arrow) in neutral position with subluxation over the lateral malleolus (long arrow) during dorsiflexion and eversion

USG of the foot and ankle was performed with a high-frequency 13 MHz probe (Philips iU22, Philips Medical Systems, DA Best, The Netherlands). The ankle is scanned via medial, lateral, and dorsal approached with patient in supine position. The patient is positioned prone to evaluate the posterior ankle and the tendo-Achilles complex. Foot is scanned via dorsal and plantar approaches. The lumps and bumps are scanned in the axial and longitudinal planes. Color and power Doppler are used to assess the vascularity of a lump and the inflammatory response. The adjacent joints, ligaments, and tendons are assessed dynamically as well.[1–3,7] Passive and active movements of the joints are performed while examining the appropriate tendons; this enables the operator to appreciate subluxation, dislocation, and tears better [Figure 1].

We present the characteristic USG findings of various lumps and bumps of the foot and ankle.

### Ganglion

**Figure 2:**
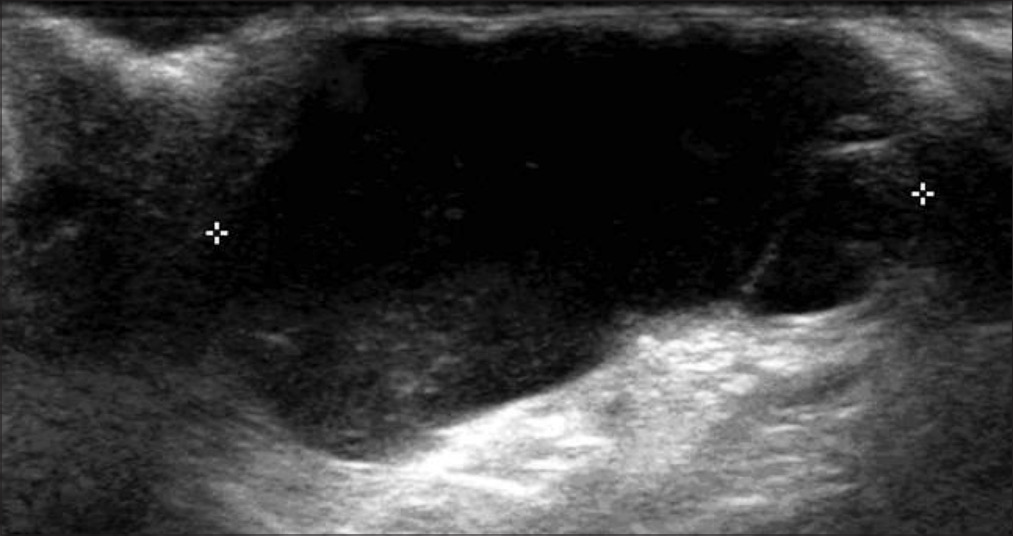
Ganglion. Longitudinal USG shows a multiloculated, anoechoic lesion (arrow) with posterior acoustic enhancement

This is seen as a well-defined, anechoic cystic lesion containing debris and lying close to the tendon sheath or joint capsule. Septations are noted in a complex ganglion, with echoes within the cyst. Color and power Doppler may demonstrate mild peripheral vascularity [Figure 2].

### Vascular malformation

**Figure 3 (A,B):**
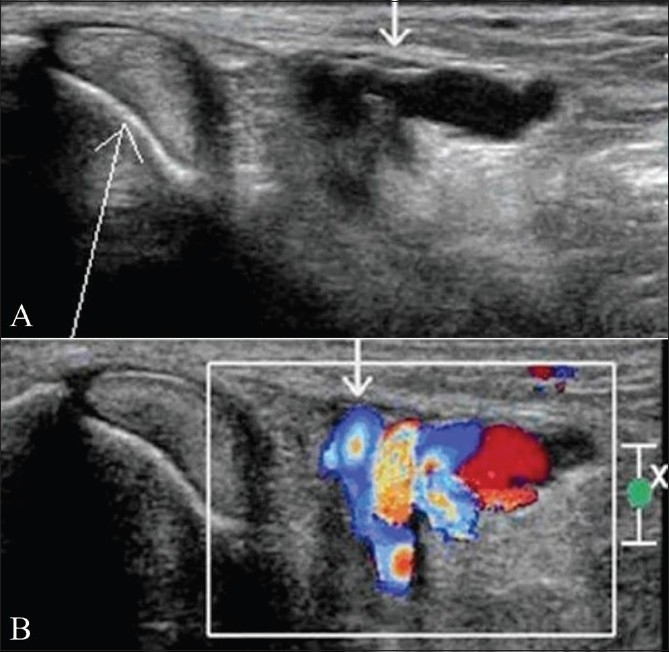
Vascular malformation in the tarsal tunnel causing compressive posterior tibial neuropathy. Transverse gray-scale USG (A) shows dilated and tortuous vessels (small arrow) with flow on color Doppler (B) in the right tarsal tunnel adjacent to the tibialis posterior tendon (long arrow)

Vascular malformations are seen as well-defined lesion with mixed echogenicity and tortousity. Color and power Doppler demonstrate intense vascularity [Figure 3]. The adjacent tendon and tendon sheaths are normal in appearance. Sometimes, the presence of small speckles of calcification (phleboliths) makes it easier to diagnose.

### Bursitis

**Figure 4:**
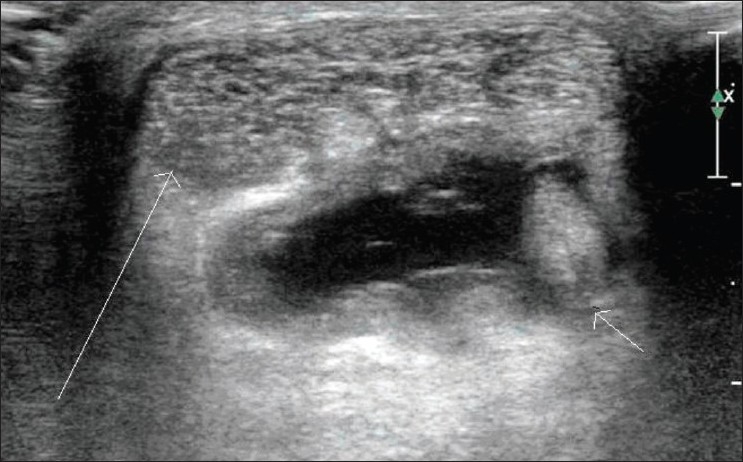
Retrocalcaneal bursitis. Transverse USG shows a fluiddistended retrocalcaneal bursa (small arrow), deep to the Achilles tendon (long arrow)

**Figure 5:**
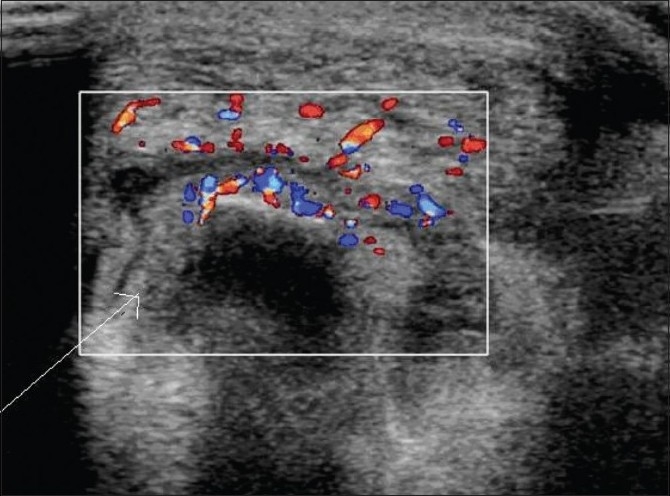
Retrocalcaneal bursitis. Transverse color Doppler USG shows intense vascularity in the wall of a distended retrocalcaneal bursa (long arrow)

Anechoic fluid within a normal bursa, with or without increase in bursal wall thickness, is seen in acute bursitis. The wall of the bursa is thickened in chronic bursitis. Hemorrhage within the bursa results in septations and calcification. Color and power Doppler may show increased vascularity. The presence of gas, along with inflammatory changes within the bursa, is suspicious of superadded infection [Figures 4 and 5].

### Tenosynovitis

**Figure 6 (A,B):**
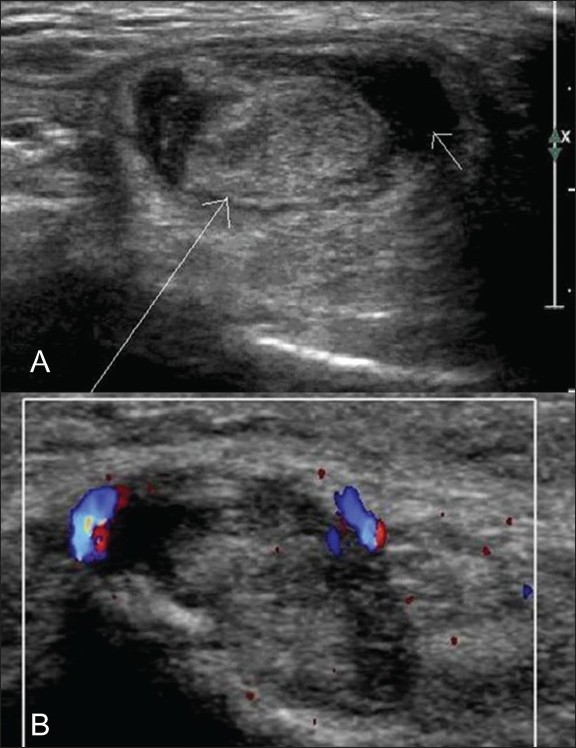
Tenosynovitis. Transverse gray-scale (A) and color Doppler (B) USG shows an edematous tibialis posterior (long arrow) with a small amount of fluid (small arrow in A) in the tendon sheath and increased vascularity on color Doppler

The tendon is enlarged and the tendon sheath is distended due to anechoic fluid. Hyperemia is noted around the tendon and within the synovium on both color and power Doppler [Figure 6]. It is advisable to search for associated retrocalcaneal bursitis in patients with Achilles tendinosis.[8]

### Tendinopathy

**Figure 7:**
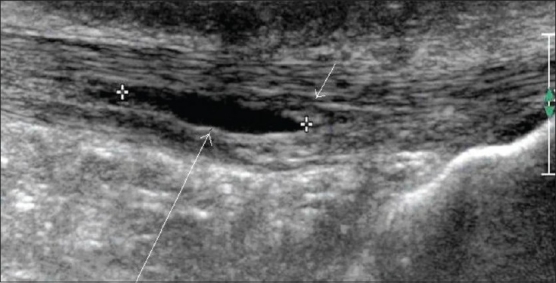
Tendinopathy. Longitudinal USG shows Achilles tendinopathy with intrasubstance cystic degeneration (arrow)

The involved tendon appears enlarged, with loss of echogenicity. Chronic tendinopathy is associated with atrophy of the tendon. Areas of calcification and cystic changes may also be associated with tendinopathy [Figure 7]. The inflamed tendon shows localized or diffuse areas of hypoechogenicity with small vessels within.[9] In the leg, the middle third of the Achilles tendon is the usual site of involvement.[10] A rupture of a tendon may also present as a lump.[10]

### Tendon subluxation

The flexor, extensor, and peroneal tendons are held in place with retinaculae. Rupture of these retinaculae may result in subluxation or dislocation of tendons. Dynamic USG enables one to interrogate this effectively. A subluxed or dislocated tendon (e.g., the tendon of the peroneus brevis) may present as a lump around the ankle [Figure 1].

### Osteophytes

**Figure 8:**
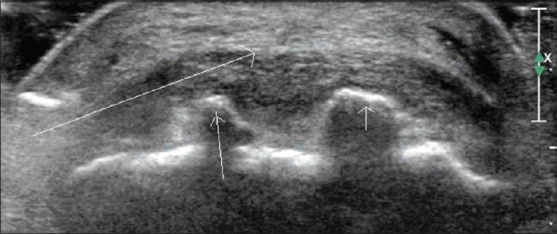
Osteophytes. Transverse USG shows osteophytes (small arrows) at the insertion of the Achilles tendon (long arrow)

These appear as areas of hyperechogenicity with posterior acoustic shadowing adjacent to the joints and continuous with the bone surface. Comparison with adjacent joints and irregularity of the joint supports the diagnosis [Figure 8].

### Morton neuroma

**Figure 9:**
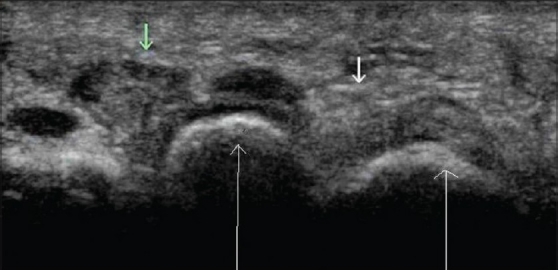
Morton's neuroma. Transverse USG shows a mixed echogenic lesion in the 2nd web space. (small green arrow), normal web space(small arrow on the right) and metatarsal heads (long arrows)

These are seen as areas with predominantly low echogenicity with minimal or no vascularity. Compression of the metatarsal heads displaces these neuromas. This maneuver helps to identify small neuromas [Figure 9].

### Foreign body granuloma

**Figure 10:**
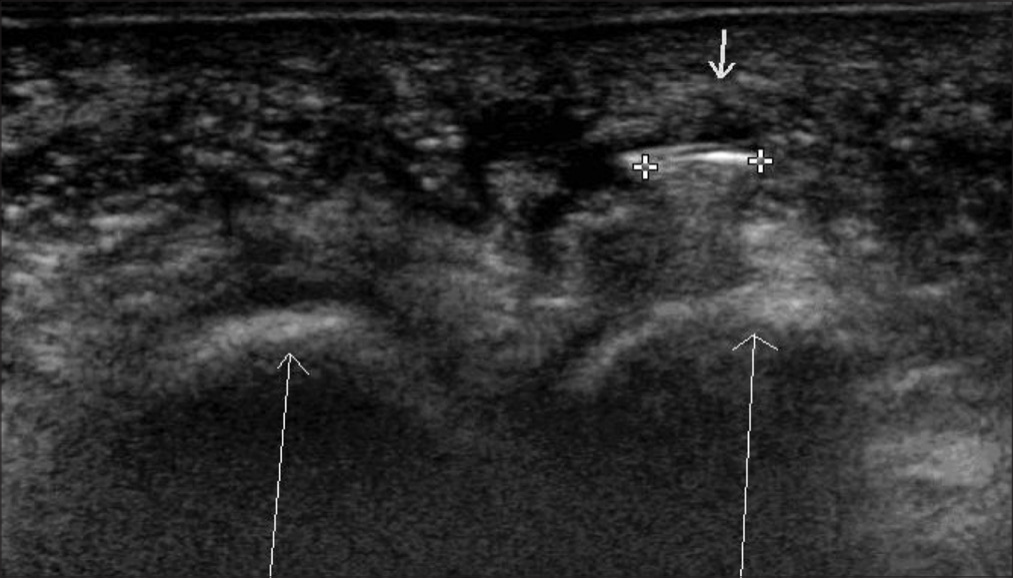
Foreign body. Transverse USG shows a hyperechoic foreign body (small arrow) in the plantar aspect of the foot. Note the metatarsal heads (long arrows)

Foreign bodies are seen as hyperechoic areas with or without posterior acoustic enhancement. Small hypoechoic areas, consistent with fluid or granulation tissue, may be seen in the vicinity [Figure 10].

### Tumors

**Figure 11:**
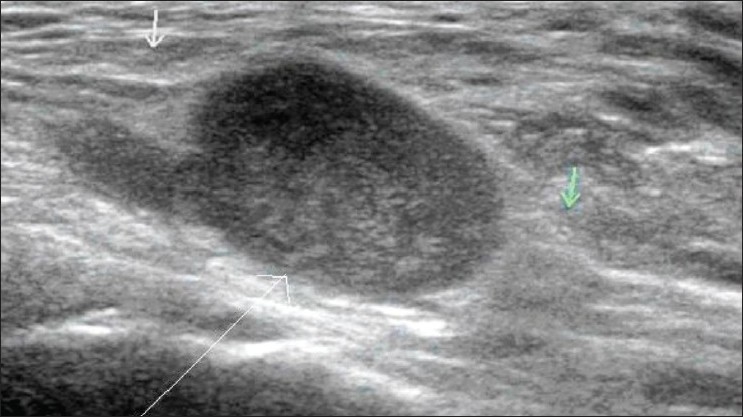
Schwannoma. Longitudinal USG shows a mixed echogenic lesion (long arrow) in relation to the posterior tibial nerve (small arrows)

Varied appearances are noted in tumors of the foot and ankle. Mixed hypo- and hyperechogenic lesions may be seen. Neurogenic tumors present as areas of varied echogenicity in relation to the nerves [Figure 11].

### Plantar fibroma

**Figure 12 (A,B):**
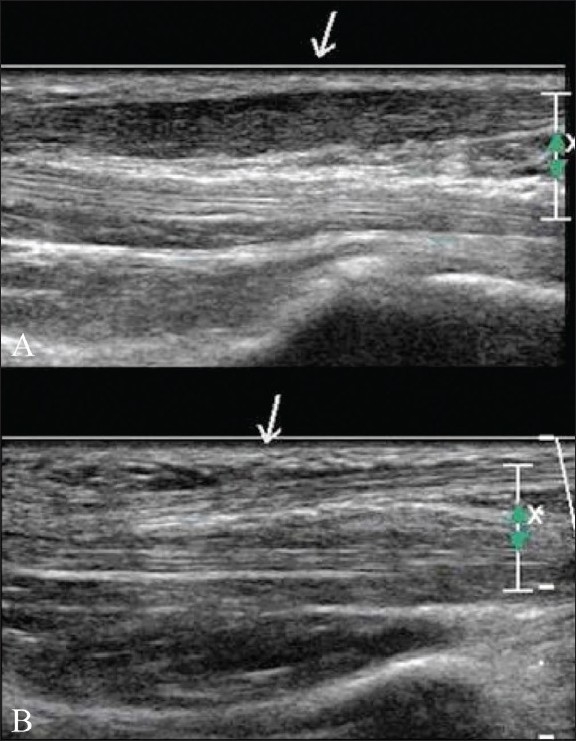
Plantar fibroma. Longitudinal USG of the left (A) and right (B) plantar fascia shows a well-defined hypoechoic lesion in the left plantar fascia (arrow in A). Note the normal right plantar fascia (arrow in B)

A plantar fibroma is seen as a well-defined hypoechoic area within the plantar fascia [Figure 12].

### Accessory bones

**Figure 13:**
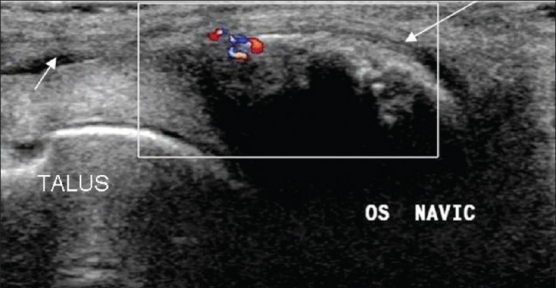
Accessory navicular. Longitudinal oblique USG of the ankle shows an accessory navicular bone/os naviculare (long arrow) at the insertion of the tibialis posterior tendon (small arrow)

These appear as areas of increased echogenicity with posterior acoustic shadowing. Knowledge of the usual anatomical sites of accessory ossicles and sesamoids is essential to make this diagnosis [Figure 13].
